# Immature Spinal Locomotor Output in Children with Cerebral Palsy

**DOI:** 10.3389/fphys.2016.00478

**Published:** 2016-10-25

**Authors:** Germana Cappellini, Yury P. Ivanenko, Giovanni Martino, Michael J. MacLellan, Annalisa Sacco, Daniela Morelli, Francesco Lacquaniti

**Affiliations:** ^1^Centre of Space Bio-medicine, University of Rome Tor VergataRome, Italy; ^2^Laboratory of Neuromotor Physiology, Istituti di Ricovero e Cura a Carattere Scientifico (IRCCS) Santa Lucia FoundationRome, Italy; ^3^School of Kinesiology, Louisiana State University, Baton RougeLA, USA; ^4^Department of Pediatric Neurorehabilitation, Istituti di Ricovero e Cura a Carattere Scientifico (IRCCS) Santa Lucia FoundationRome, Italy; ^5^Department of Systems Medicine, University of Rome Tor VergataRome, Italy

**Keywords:** cerebral palsy, abnormal development, basic muscle activation patterns, spinal locomotor output, gait

## Abstract

Detailed descriptions of gait impairments have been reported in cerebral palsy (CP), but it is still unclear how maturation of the spinal motoneuron output is affected. Spatiotemporal alpha-motoneuron activation during walking can be assessed by mapping the electromyographic activity profiles from several, simultaneously recorded muscles onto the anatomical rostrocaudal location of the motoneuron pools in the spinal cord, and by means of factor analysis of the muscle activity profiles. Here, we analyzed gait kinematics and EMG activity of 11 pairs of bilateral muscles with lumbosacral innervation in 35 children with CP (19 diplegic, 16 hemiplegic, 2–12 years) and 33 typically developing (TD) children (1–12 years). TD children showed a progressive reduction of EMG burst durations and a gradual reorganization of the spatiotemporal motoneuron output with increasing age. By contrast, children with CP showed very limited age-related changes of EMG durations and motoneuron output, as well as of limb intersegmental coordination and foot trajectory control (on both sides for diplegic children and the affected side for hemiplegic children). Factorization of the EMG signals revealed a comparable structure of the motor output in children with CP and TD children, but significantly wider temporal activation patterns in children with CP, resembling the patterns of much younger TD infants. A similar picture emerged when considering the spatiotemporal maps of alpha-motoneuron activation. Overall, the results are consistent with the idea that early injuries to developing motor regions of the brain substantially affect the maturation of the spinal locomotor output and consequently the future locomotor behavior.

## Introduction

Cerebral palsy (CP) is defined as a group of permanent disorders that affect the development of movement and posture attributed to non-progressive disturbances that occurred in the developing fetal or infant brain (Bax et al., 2005; Rosenbaum et al., 2014). Seventy percent or more of children with CP experience problems with walking (Hutton and Pharoah, 2002). The mechanisms and functional significance of early maturation of pattern generation networks and its interrelation with cerebral dysfunction are of crucial importance in understanding the pathophysiology of CP (Yang et al., 2013). There is now a growing interest in quantifying dynamic EMGs and kinematic patterns as predictive indicators of motor function in CP. For instance, the specific features of spontaneous movements provide useful information for predicting CP in very young infants (Hadders-Algra, 2014; Kanemaru et al., 2014). In spite of the existence of various classifications of early sensorimotor pathologies (Rosenbaum et al., 2014), detailed information about maturation of the spinal locomotor output in children with CP is much needed for both clinical diagnosis and assessment of the efficacy of gait rehabilitation.

Human locomotion is the result of a complex interplay between supraspinal, sensory, and spinal pattern generators signals. The final neural output is represented by the spatiotemporal modulation of alpha-motoneuron (MN) activity, which can be assessed by mapping the activity patterns from a large number of simultaneously recorded muscles onto the anatomical rostrocaudal location of the MN pools in the spinal cord (Yakovenko et al., 2002; Ivanenko et al., 2006b; Wenger et al., 2016), and by decomposing the coordinated muscle activation profiles into a small set of common factors as a means to look backward from the periphery to the CNS (Davis and Vaughan, 1993; Bizzi et al., 2000; Ivanenko et al., 2006a; Cheung et al., 2012; Chvatal and Ting, 2013; Danner et al., 2015; Giszter, 2015). Using these approaches, it has been shown that the development of locomotion in typically developing (TD) children involves a functional reorganization of the spinal locomotor output. In particular, the number of basic muscle activation patterns increases from the neonate to the toddler (Dominici et al., 2011; Lacquaniti et al., 2012a), and the temporal dynamics of MN activation in lumbar and sacral spinal segments undergoes structured functional changes during early locomotor development (Ivanenko et al., 2013a). How these developmental processes are altered in children with CP remains largely unknown.

There are only a few studies that attempted to evaluate the spatiotemporal organization of the spinal locomotor output in CP (Steele et al., 2015; Tang et al., 2015; Shuman et al., 2016) suggesting that individuals with CP may use a simplified control strategy (fewer synergies) compared with unimpaired individuals. However, a more detailed and thorough consideration of multi-muscle coordinated patterns is needed (Damiano, 2015). While several gait features and sensory feedback impairments have previously been documented in CP, it is less clear whether the spinal motor patterns undergo a functional reorganization similar to that occurring during normal development and how they evolve into mature walking.

The aim of the present study was to assess the locomotor patterns in children with diplegic and hemiplegic CP aged between 2 and 12 years, and compare them with those in TD children. Recent work from our group has placed a special focus on the spatiotemporal characteristics of the spinal locomotor output (Dominici et al., 2007, 2011; Ivanenko et al., 2013a; Martino et al., 2015). Some previous studies concluded that the locomotor patterns of older children with cerebral palsy show similarity of the early stages of gait development in healthy children (Leonard et al., 1991; Berger, 1998; Meyns et al., 2012), and these have reported mainly the characteristics and the degree of coactivation of only a few muscles. Given that the small number of recorded muscles limits our conclusions on the spatiotemporal structure of muscle activity patterns (Steele et al., 2013; Zelik et al., 2014; Damiano, 2015), the rationale of this study was to examine the modular organization and the temporal dynamics of MN activation in lumbar and sacral spinal segments based on EMG activity of 11 pairs of bilateral muscles with lumbosacral innervation. While the general hypothesis of delayed maturation has been previously put forward (Forssberg, 1999), our study is the first demonstration of the spinal segmental output, characteristics of these patterns and their progression with age. Overall, the findings indicate that a perinatal brain damage prevents maturation of locomotor activity in children with CP and thereby affects the locomotor behavior.

## Materials and methods

### Participants and protocol

Thirty-three children, born at term, clinically defined as TD by their pediatrician (age range 1.0-11.8 years; individual and average characteristics are listed in Table 1) were recruited by word of mouth. These children belonged to two groups, the first group including seven younger toddlers at their first steps aged 1–1.2 years and a second group of 26 older children aged 2.1–12 years. Sixteen children with a clinical diagnosis of hemiplegia due to CP (age range 2.3–11.8 years; 10 right and 6 left hemiplegia), and 19 children with diplegic CP (age range 2.3–11.1 years) were recruited from the Department of Pediatric Neurorehabilitation of IRCCS Santa Lucia Foundation (Table 2). Twenty-three children with CP (66%) had been born preterm (<37 post-menstrual age). The Ethics Committee of IRCCS Santa Lucia Foundation approved the study procedures that adhered to the Declaration of Helsinki for medical research involving human participants, and informed written consent was obtained from the parents of all (TD and CP) children. Because the definition of CP is usually not etiologic but functional, the inclusion criteria of hemiplegia/diplegia in this study were also based on the assessment of motor functioning. Accordingly, hemiplegia has been defined as a neuromuscular disorder that involves one-half of the body (most affected side) in the frontal plane while the other half is normal or near normal (least affected side).

**Table 1:** **Characteristics of typically developing (TD) subjects and the number of analyzed strides and range of speeds**.

**Subjects**	**Age, years**	**Gender**	**GA, wk**	**BW, gr**	**wo, mo**	**Strides, n**	**Speed, km/h**
TD1	1.0	F	40	2670	11	80	0.7–1.3
TD2	1.0	F	38	3500	12	27	0.1–2.9
TD3	1.1	F	39	3400	12	41	0.1–2.9
TD4	1.1	F	39	3100	13	150	0.2–1.4
TD5	1.2	F	40	3000	12	74	0.6–1.4
TD6	1.2	M	38	3040	13	63	0.1–1.2
TD7	1.2	M	38	3430	13	93	0.6–1.4
TD8	2.1	F	39	3000	12	48	1.1–2.8
TD9	2.6	F	38	2900	16	81	1.4–4.3
TD10	2.8	M	38	3000	15	65	1.5–4
TD11	2.9	M	39	2800	12	87	1.6–2.4
TD12	3.0	F	41	2750	15	80	1.5–3.2
TD13	3.3	M	39	3450	14	75	0.8–2.6
TD14	4.0	M	38	2900	12	83	0.8–4.2
TD15	4.2	M	39	3000	11	81	1.2–3.9
TD16	4.3	M	38	2700	12	152	1–4.5
TD17	4.6	F	40	3000	12	101	1.9–3.4
TD18	4.9	F	38	3000	15	105	1.6–4.9
TD19	5.3	F	38	2900	16	61	2.4–4.7
TD20	5.7	F	41	2750	15	68	2.3–3.9
TD21	5.8	M	39	3100	12	64	2–5.1
TD22	5.8	F	38	3100	12	59	2.4–4
TD23	6.1	M	38	2700	12	37	2.2–4.6
TD24	6.2	F	30	1500	11	69	2.2–5.7
TD25	6.4	F	40	3800	12	61	1.9–4.1
TD26	6.8	M	38	3000	12	83	1.8–3.9
TD27	7.1	M	39	2900	12	54	1.7–4.4
TD28	7.2	M	38	2700	12	54	1.7–4.5
TD29	7.6	F	38	3100	12	68	1.6–4
TD30	8.8	F	38	3100	15	66	2.6–4.4
TD31	10.3	F	39	3900	12	30	1.6–4.9
TD32	10.8	M	39	3000	12	60	1.7–3.5
TD33	11.8	F	38	3100	12	48	2.2–4.9
Mean (±SD)			38.5 ± 1.8	3009 ± 401	12.8 ± 1.5	72 ± 28	

GA, gestational age at birth; BW, birth weight; wo, walking onset.

**Table 2:** **Characteristics of children with cerebral palsy and the number of analyzed strides and range of speeds**.

**Subjects**	**TCP**	**Side**	**Gender**	**Age, years**	**Brain lesion**	**AFO**	**GMFC-s**	**GMFM**	**GA, wk**	**BW, gr**	**wo, mo**	**Strides, n**	**Speed, km/h**
CP1	HE	L	M	2.3	Right hemispheric wm + basal ganglia		2	45.59	30	1520	26	45	1.1–2.9
CP2	HE	L	F	2.7	PVL		1	80.37	30	1215	15	63	1.2–4.3
CP3	HE	R	F	2.8	Bilat. cortical–subcortical		1	95.70	38	3650	18	35	1.3–2.7
CP4	HE	L	M	3.0	Right hemisphere	×	1	85.04	31	1580	30	58	1.1–2.8
CP5	HE	R	M	3.8	Left wm + basal ganglia		1	94.52	31	1400	15	92	1–4
CP6	HE	L	M	4.7	PVL	×	1	88.71	38	2820	12	14	0.7–1.8
CP7	HE	R	F	5.0	PVL	×	1	70.51	40	3200	14	33	1.6–2.6
CP8	HE	R	M	5.0	Left subcortical		1	97.02	34	2770	22	59	2.1–2.9
CP9	HE	L	F	5.2	Right hemisphere		1	95.40	40	4400	24	37	1.8–3.7
CP10	HE	L	M	5.5	Right cortical-subcortical		1	78.73	39	3810	16	86	3–4.3
CP11	HE	R	F	6.8	PVL		1	98.93	37	2380	12	32	2.7–4.8
CP12	HE	R	M	8.4	Left cortical		1	99.44	36	3240	21	25	2.1–3.2
CP13	HE	R	F	9.9	Left wm + basal ganglia	×	2	84.91	31	1200	30	67	2.4–4.9
CP14	HE	R	M	11.4	PVL		1	95	36	2000	24	25	2.7–4.6
CP15	HE	R	M	11.8	Left hemisphere		2	83.30	35	1700	35	20	1.9–3
CP16	HE	R	M	11.8	Left hemisphere		1	97.86	38	3580	13	40	1.7–2.9
CP17	DI		M	2.3	PVL	×	1	62.62	30	1450	26	27	0.1–0.8
CP18	DI		M	2.5	PVL	×	1	80	32	2000	29	29	0.9–2.7
CP19	DI		F	2.6	PVL		1	80.37	29	1215	15	63	1.2–4.3
CP20	DI		M	2.7	PVL		1	86.12	39	1300	20	48	0–2
CP21	DI		F	2.8	PVL	×	1	72.81	27	1200	21	32	0.3–0.7
CP22	DI		M	3.0	PVL		1	80.7	29	1650	20	76	0.9–2.6
CP23	DI		M	3.1	PVL		1	87.86	31	1704	19	84	2–4.4
CP24	DI		M	3.2	PVL		1	76.06	31	1900	20	60	1.9–3.3
CP25	DI		F	3.3	PVL		1	82.88	34	2065	33	34	1.4–2
CP26	DI		M	3.4	PVL	×	1	89.88	41	3676	18	30	1.4–2.3
CP27	DI		M	3.8	PVL		1	94.52	31	1400	15	38	1–4
CP28	DI		M	4.2	PVL		3	59.04	29	780	36	45	0.8–1.9
CP29	DI		F	5.2	PVL		2	89.97	32	2800	20	25	2–3.8
CP30	DI		F	5.4	PVL	×	2	80.42	38	3110	36	90	0.8–2.6
CP31	DI		F	6.1	PVL		1	85	30	1500	30	102	1.8–3.9
CP32	DI		M	6.8	PVL		2	88.48	39	3100	30	40	1.5–2.3
CP33	DI		M	8.8	PVL		1	88	33	1800	30	61	2.7–3.7
CP34	DI		M	10.5	PVL		2	85.11	30	2100	30	45	1.4–3
CP35	DI		F	11.1	PVL		1	80.4	37	3150	96	88	0.9–2
Mean (±SD)							1.3 ± 0.5	84 ± 12	33.9 ± 4	2244 ± 942	24.9 ± 14.3	50 ± 24	

TCP, type of CP; DI, diplegia; HE, hemiplegia; side, affected side; wm, white matter lesions; PVL, periventricular leukomalacia; AFO, ankle-foot orthosis; GMFC-s, Gross Motor Function Classification System; GMFM, Gross Motor Function Measure; GA, gestational age at birth; BW, birth weight; wo, walking onset.

Clinical diagnosis was based on the predominant type of motor impairment and classified according to the criteria proposed by Himmelmann et al. (2005). The children were divided into two groups according to the type of CP: diplegic and hemiplegic. CP diagnosis was confirmed according to medical history, brain magnetic resonance results and clinical examination. Clinical characteristics such as gestational age at birth, birth weight and documented type of brain lesion for the children diagnosed with CP are presented in Table 2. Most diplegic CP children had a periventricular white matter injury (damage of white matter, often termed “periventricular leucomalacia”), while most children with hemiplegia had gray matter lesions, usually accompanied by different lesions in other brain areas specific for each child, which was in accordance with prevalence and etiology of CP.

Motor function was evaluated using the Gross Motor Function Classification System (GMFCS, (Palisano et al., 1997, 2006). The clinical characteristics of the children are summarized in Table 2. All children with CP were classified between level I and III of the GMFCS. Inclusion criteria were: GMFCS≤3 and CFCS (Communication Function Classification System, Hidecker et al., 2011) ≤3. Exclusion criteria were: lower extremity orthopedic surgery within the past year or botulinum toxin A injections within the past 4 months. Furthermore, to assess motor function for each child with CP, we used the Gross Motor Function Measure (GMFM), a standardized observational instrument of 88 items grouped into 5 domains, related primarily to postural and locomotor abilities. It measures gross motor function, during lying and rolling, crawling and kneeling, sitting, standing, and walk-run-jump activities with a scale of 0–100 (Russell et al., 1989, 2000). The assessments of GMFCS and GMFM were carried out by experienced physiotherapists in accordance with the manuals available for both instruments. All participants were able to understand the instructions, to walk in an autonomous manner for the duration of the experiment, and to perform the test properly. If children with CP used an ankle-foot orthosis for daily activities, it was removed for the duration of the experiment.

Experiments were performed in the Laboratory of Neuromotor Physiology, IRCCS Santa Lucia Foundation by the gait analysis experts, in the presence of a neuropediatrician, physiotherapist, and one or both parents of the child. Children were asked to walk barefoot for ~8 m in a straight path at a self-selected speed in a large (11 × 14 m) experimental room. For the recording of the first steps in TD toddlers, one experimenter (or parent) initially held the toddler by hand and then moved forward, leaving the toddler's hand and encouraging the toddler to walk unsupported on the floor toward another experimenter (or parent).

### Data recording

**Figure 1:**
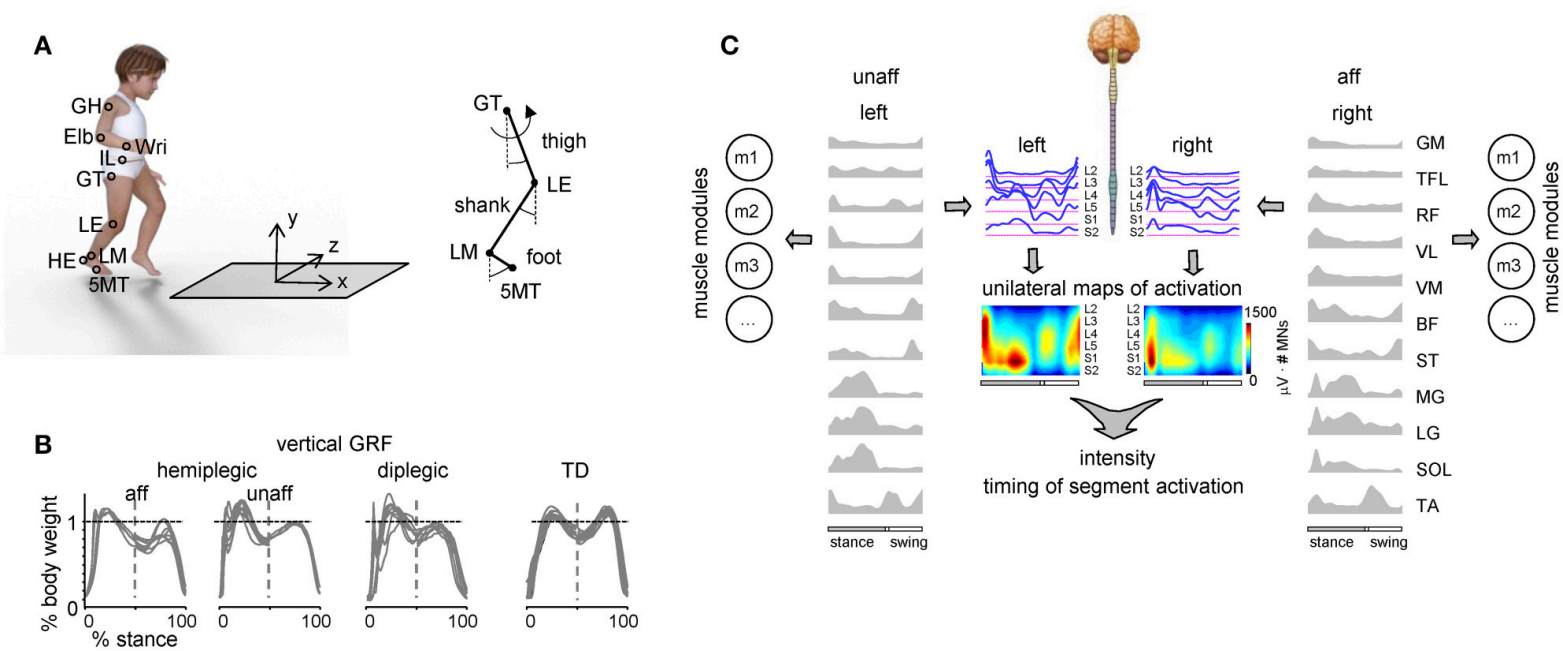
**Experimental setup and recorded spatiotemporal gait parameters. (A)** Schematic view of the experimental setup and recorded body landmarks and angles. Elb, elbow; GH, gleno-humeral joint; HE, heel; IL, ilium; LE, lateral femur epicondyle; LM, lateral malleolus; Wri, wrist. **(B)** Examples of vertical ground reaction forces in one representative child with hemiplegia (9.9 years), one diplegia (6.1 years) and one TD (11.8 years) child. The data from several strides were superimposed. Since children typically made more than one foot placement on the force plate (so that we could not isolate the force of one leg throughout the whole stance phase), the ground reaction forces in the first and second half of the stance were taken from different strides (and separated by the vertical dashed line). **(C)** Example of the spatiotemporal patterns of muscle and motoneuron activity along the rostrocaudal axis of the spinal cord during walking in one child with hemiplegic. Recorded EMG waveforms were used to reconstruct muscle modules (m_i_) and output pattern of each segment of each side of the lumbosacral enlargement (blue curves). Same pattern is plotted in a color scale (right calibration bar) using a filled contour plot (middle). Patterns are plotted vs. the normalized gait cycle.

We recorded the kinematics bilaterally at 100 Hz by means of a Vicon-612 system (Oxford, UK) with 9 cameras placed around the walking path. Infrared reflective markers were attached on each side of the participant to the skin overlying the following landmarks: gleno-humeral joint (GH), elbow (Elb), wrist (Wri), ilium (IL), greater trochanter (GT), lateral femur epicondyle (LE), lateral malleolus (LM), heel (HE), and fifth metatarso-phalangeal joint (5MT) (Figure 1A). In a subset of children (six hemiplegic, 10 diplegic, 16 TD), we also recorded ground reaction forces at 1000 Hz by means of a force platform (0.9 × 0.6 m; 9287B; Kistler, Zurich, Switzerland) (Figure 1A).

Electromyographic (EMG) activity was recorded by means of surface electrodes from 22 muscles simultaneously. The following 11 muscles were recorded from each body side: BF, biceps femoris (long head); GM, gluteus maximus; LG, gastrocnemius lateralis; MG, gastrocnemius medialis; RF, rectus femoris; SOL, soleus; VL, vastus lateralis; ST, semitendinosus; TA, tibialis anterior; TFL, tensor fascia latae; VM, vastus medialis. All EMGs were recorded at 2000 Hz using the Trigno Wireless EMG System (Delsys Inc., Boston, MA), bandwidth of 20–450 Hz, overall gain of 1000. Sampling of kinematic, ground reaction force and EMG data was synchronized.

### General gait parameters

Gait cycle was defined as the time between two successive foot–floor contacts by the same leg according to the local minima of the vertical displacement of the heel (or 5MT in case of toe-walking) marker (Dominici et al., 2007). The timing of the lift-off was determined analogously (when the 5MT marker was elevated by more than 2 cm). The touch-down and lift-off events were also verified from the ground reaction force recordings when available (i.e., the time at which the vertical force went above or below 7% of the body weight, respectively), and we found that our kinematic criteria predicted the onset and end of stance phase with an error smaller than 2% of the gait cycle duration (Dominici et al., 2007). For the analysis of locomotor patterns, the steps related to gait initiation and termination were discarded, and only those performed in the central section of the path at about constant speed were included in the analysis.

Walking speed for each stride was computed as the mean speed of the horizontal trunk movement, the latter being identified by the time course of the displacement of a virtual marker located at the midpoint between left and right ilium markers. Stride length was measured according to horizontal displacement of the foot maker (5MT). Data were time-interpolated over individual gait cycles to fit a normalized 200-point time base. The stride length and foot trajectory during swing were normalized to the limb length (L, determined by summing lengths of the thigh and shank segments) of the participants.

### Intersegmental coordination

By analyzing limb kinematic coordination, one may infer the characteristics of the developing human locomotor system (Cheron et al., 2001a,b; Dan et al., 2004; Ivanenko et al., 2007; Yang et al., 2015). Intersegmental coordination was evaluated in angular position space as previously described (Borghese et al., 1996; Bianchi et al., 1998). The body was modeled as an interconnected chain of rigid segments: GH–IL for the trunk, GH–Elb for the arm, Elb–Wri for the forearm, IL–GT for the pelvis, GT–LE for the thigh, LE–LM for the shank, and LM–5MT for the foot. The main lower limb axis was defined as GT–LM. The elevation angle of each segment in the sagittal plane (including the lower limb axis) corresponds to the angle between the segment projected on the sagittal plane and the vertical (positive in the forward direction, i.e., when the distal marker is placed anteriorly to the proximal one). In healthy adults, the temporal changes of the elevation angles at the thigh, shank, and foot covary tightly during walking (Borghese et al., 1996; Bianchi et al., 1998). When these angles are plotted in three dimensions (3D), they describe a path that can be least-squares fitted to a plane over each gait cycle. Here, we studied the intersegmental coordination (the gait loop and its associated plane) in hemiplegic, diplegic and TD children. To this end, we computed the covariance matrix of the ensemble of time-varying elevation angles (after subtraction of their mean value) over each gait cycle. The three eigenvectors u_1_–u_3_, rank-ordered on the basis of the corresponding eigenvalues, correspond to the orthogonal directions of maximum variance in the sample scatter. The first two eigenvectors u_1_ and u_2_ lie on the best-fitting plane of angular covariation. The third eigenvector (u_3_) is the normal to the plane, and defines the plane orientation. To quantify the rotation of the plane (Bianchi et al., 1998; Dominici et al., 2007), we analyzed the u_3*t*_ parameter (the direction cosine of the normal to the plane with the axis of thigh elevation).

### EMG activity and basic activation patterns

The raw EMG signals were high-pass filtered (30 Hz), demeaned, rectified and low-pass filtered with a zero-lag fourth-order Butterworth filter (10 Hz). The time scale was normalized by interpolating individual gait cycles over 200 points. Both individual muscle EMG characteristics and basic activation patterns (common features across muscle activities) were analyzed.

Basic activation patterns were extracted from the EMG envelopes (Figure 1C) using the non-negative matrix factorization algorithm (Lee and Seung, 2001; Tresch et al., 2006; Martino et al., 2015). For this analysis, the EMG signal from each muscle was normalized to its peak value across all trials for each participant. However, we also verified whether the results depended on the normalization procedure by considering non-normalized EMG signals (in μV).

The processed EMG envelopes were combined into an *m* × *t* matrix (where *m* is the number of muscles, and *t* is 200 × number of gait cycles collected across all trials). Thus, the NNMF was applied to all strides of each participant to identify the underlying basic activation patterns (*P*) in the EMG recordings. The algorithm searches for an approximate solution:

(1) $$\begin{array}{l} EMG=\sum_{i}^{n}P_{i}W_{i}+error,\;n\leq m \end{array}$$

where the measured *EMG* is modeled as a linear composition of basic activation patterns *P* (*n* × *t* matrix, where *n* is a predetermined number of basic patterns, see below) and weighting coefficients or muscle synergies *W* (*m* × *n* matrix). The *W* and *P* are estimated to minimize the root-mean-squared error between *EMG* and *P* × *W*. The factorization uses an iterative method starting with random initial values for *W* and *P*. Because the root-mean-squared error may have local minima, the best solution was selected out of 100 runs to find *W* and *P* from multiple random starting values. Each run of the NNMF was executed until a termination tolerance on change in size of the residual error or in the elements of *W* and *P* set to 10^−5^. The basic activation patterns were then segmented back into individual cycles (*n* × 200) for averaging or for individual stride analysis.

Pattern decomposition was assessed by calculating the percent of variability (or variance) accounted for (Torres-Oviedo et al., 2006):

(2) $$\begin{array}{l} VAF=sum\;of\;squared\;errors/total\;sum\;of\;squares. \end{array}$$

where the total sum of squares is taken with respect to the mean over the rows of the data matrix. To determine the minimum number of basic activity patterns *n* which best accounts for the EMG data variance, we used a method (“*Best linear fit*”) based on a linear regression procedure (d'Avella et al., 2006) by varying the number of basic patterns from 1 to 8, and selecting the smallest *n* such that a linear fit of the *VAF* vs. *n* curve had a residual mean square error less than 10^−4^.

The structure of muscle modules was compared using cluster analysis. To identify and average similar basic patterns across participants, we evaluated the optimal number of data clusters from the set of the extracted synergies *W* for each group using the Calinski-Harabasz Index (CH index, Caliñski and Harabasz, 1974). The number of clusters correspond to the number of similar patterns across participants. To match similar patterns, we slightly modified our previous algorithm (Martino et al., 2015) by evaluating the degree of similarity based on the best-matching summed scalar product of both weighting coefficients and temporal patterns normalized to the Euclidean norm, since each module involves a basic activation pattern (temporal structure) with variable weights of distribution (spatial structure) to different muscles. Briefly, we first computed the best-matching scalar products between each individual set and the mean of randomly ranked inter-individual sets. Then we iteratively updated this mean by comparing every possible combination and finding the one that maximized the total summed scalar product. If modules from one set were not matched to the modules from the mean set (summed scalar product < 0.8), we isolated the unmatched modules.

To characterize differences in the duration of EMG activity and basic patterns between groups, we computed the full width at half maximum (*FWHM*). *FWHM* was calculated as the sum of the durations of the intervals in which the rectified EMG or the basic activation patterns (after subtracting the minimum throughout the gait cycle) exceeded half of their maximum. To characterize differences in the timing of EMG bursts and basic activation patterns, we computed the center of activity (*CoA*). These parameters were calculated over individual strides and then averaged across cycles.

The *CoA* during the gait cycle was calculated using circular statistics (Batschelet, 1981) as the angle of the vector (1st trigonometric moment) in polar coordinates (polar direction denoted the phase of the gait cycle, with angle θ that varies from 0 to 360°) that points to the center of mass of that circular distribution using the following equations:

(3) $$\begin{array}{r} A=\overset{200}{\underset{t=1}{\sum }}\left(\cos\theta_{t}\times P_{t}\right) \end{array}$$

(4) $$\begin{array}{r} B=\overset{200}{\underset{t=1}{\sum }}\left(\sin\theta_{t}\times P_{t}\right) \end{array}$$

(5) $$\begin{array}{r} CoA=\tan^{-1}\left(B/A\right) \end{array}$$

The *CoA* was chosen because it was impractical to reliably identify a single peak of activity in the majority of muscles, especially in CP children. It can only be considered as a rough estimate of the timing of EMG bursts, because averaging between distinct foci of activity may identify a poorly representative *CoA* in the intermediate zone. Nevertheless, *CoA* can be helpful to understand if the distribution of muscular activity remains unaltered across different groups of children and muscles.

To characterize the reciprocal dynamics of muscle activation, we also computed the antagonist co-activation index (*CI*). The *CI* was assessed between the thigh (mean activity of quadriceps RF-VL-VM vs. hamstring BF-ST) and calf (mean activity of triceps MG-LG-SOL vs. TA) antagonistic muscle groups using the following formula (Rudolph et al., 2000; Martino et al., 2014):

(6) $$\begin{array}{l} CI=\frac{\overset{200}{\underset{j=1}{\sum }}\left[\left(EMG_{H}\left(j\right)+EMG_{L}\left(j\right)/2\right)\right]\times \left(EMG_{L}\left(j\right)/EMG_{H}\left(j\right)\right)}{200} \end{array}$$

where *EMG*_*H*_ and *EMG*_*L*_ represent the highest and the lowest activity between the antagonist muscle pairs (EMG activity for each muscle was normalized to its maximum value). In order to have a global measure of the co-activity level, the *CI* was then averaged over the entire gait cycle (*j* = 1:200). This method provided a sample-by-sample estimate of the relative activation of the pair of muscles as well as the magnitude of the co-contraction over the entire cycle. Using this equation, high co-contraction values represent a high level of activation of both muscles, whereas low co-contraction values indicate either low level activation of antagonists, or a high level activation of one muscle along with low level activation of the other muscle in the pair (Rudolph et al., 2000).

### Motor output of the spinal segments

To characterize the spatiotemporal organization of the total motor output, the recorded averaged profiles of EMG-activity were mapped onto the rostrocaudal location of MN-pools in the human spinal cord derived from published literature (Figure 1C). This approach provides an interpretation of the motor pool activation at a segmental level rather than at the individual muscle level (Yakovenko et al., 2002; Grasso et al., 2004; Ivanenko et al., 2006b; Monaco et al., 2010; Wenger et al., 2016). It can be used to characterize the spinal locomotor output by considering relative intensities, spatial extent, and temporal structure of the spinal motor output. Because the method has been thoroughly documented in several previous papers (Grasso et al., 2004; Ivanenko et al., 2006b, 2013a; Cappellini et al., 2010; MacLellan et al., 2012; La Scaleia et al., 2014), here we describe it only briefly. In this study we used the myotomal charts of Kendall et al. (2005). Despite likely anatomical variability, the data from these charts appear sufficiently robust for the spatial resolution currently available based on EMG recordings from multiple lower limb muscles. In general, each muscle is innervated by several spinal segments, and each segment supplies several muscles. To reconstruct the motor-pool output pattern of any given spinal segment *S*_*j*_ of the lumbosacral segments (L2-S2) most active during locomotion, all rectified EMG-waveforms corresponding to that segment were averaged using appropriate weighting coefficients according to the following equation (La Scaleia et al., 2014):

(7) $$\begin{array}{l} S_{j}=\frac{\overset{m_{\text{j}}}{\underset{i=1}{\sum }}\left(\frac{k_{ji}}{l_{i}}\times {EMG}_{i}\right)}{\overset{m_{j}}{\underset{i=1}{\sum }}\left(\frac{k_{ji}}{l_{i}}\right)}\times {MN}_{j} \end{array}$$

where *EMG*_*i*_ represents the subject-specific envelope of muscle activity (in μV), *k*_*ji*_ is a weighting coefficient for the *i*-th muscle (to signify if the *j*-th spinal level is a major, *k*_*ji*_ = 1, or minor, *k*_*ji*_ = 0.5, MN source), *m*_*j*_ is the number of muscles innervated by the *j*-th spinal segment, and *l*_*i*_ is the total number of spinal levels that innervate the *i*-th muscle, again accounting for major and minor sources. Thus, the fractional part of Equation (7) can range in value from 0 (inactive) to maximum activation of that spinal segment. To account for size differences in MN pools at each spinal level, this fractional activity value was then multiplied by the segment-specific number of MNs (*MN*_*j*_), taken from Tomlinson and Irving (1977). This MN pool size normalization primarily affects the boundary segments L2 and S2, which contain 2–3 times fewer MNs than the other segments.

These waveforms were compared among the groups of children we studied. To visualize a continuous smoothed rostrocaudal spatiotemporal activation of the spinal cord, we used a filled contour plot that computes isolines calculated from the activation waveform matrix and fills the areas between the isolines using separate colors. In order to compare the general spatiotemporal features of the lumbosacral enlargement activation in different groups of participants, and the relative activation of lumbar vs. sacral segments in particular, we also computed the timing of the maximal activation throughout the gait cycle, and the ratio between the mean motoneuron activity in the dominant lumbar (sum of activity from L3 and L4) and sacral (sum of activity from S1 and S2) segments.

Motor pools are fairly stable in longitudinal spatial placement across individuals in both children and adults (Sharrard, 1955; Kendall et al., 2005), though some minor anatomical deviations from the norm in single individuals have been documented (Phillips and Park, 1991; Stewart, 1992). To verify whether the main features of the spinal maps (the timing of the maximal activation throughout the gait cycle and intensity) remain insensitive to potential small individual variations in muscle innervation, we also computed the spinal maps (1) assuming a weighting coefficient in the Equation (7) either 0 or 1 (instead of 0.5), and (2) using the Sharrard data (Sharrard, 1964) instead of the Kendall chart. In the latter case, however, we did not normalize for size differences in MN pools at each spinal level (Tomlinson and Irving, 1977) because these data were not provided by Sharrard. Nevertheless, the Sharrard data table for innervation approximates the proportion of total muscle activation attributable to each segment (by taking multiple slices within each spinal segment), instead of assuming equal proportions in all segments (Ivanenko et al., 2006b).

### Statistics

Descriptive statistics included the calculation of the mean and standard deviation (SD). One-way ANOVA was used to evaluate the effect of group. If ANOVA resulted in a significant effect, then a Tukey HSD (Honestly Significant Difference) *post-hoc* test was used to detect differences between groups. Statistics on correlation coefficients was performed on the normally distributed, Z-transformed values. Statistical analysis of circular data (Batschelet, 1981) was used to characterize the mean orientation of the normal to covariance plane and *CoA* (see preceding text) and their variability across steps. Reported results are considered significant for *p* < 0.05.

## Results

### General gait parameters

Participants usually performed several steps at approximately constant speed (see Tables 1, 2 for the number of recorded steps). The results of TD children were analyzed separately for the two age groups: toddlers (1–1.2 years) and older children age-matched to children with CP (2–12 years). The rationale for investigating a separate group of younger TD toddlers (not age-matched with children with CP) is that some gait characteristics (flat or forefoot foot placements, knee flexion in mid-stance) in children with CP may remain similar to those in toddlers at the onset of independent walking (Ivanenko et al., 2007) without fully evolving in more mature gait (Berger et al., 1984; Leonard et al., 1991). Therefore, we placed a particular focus on comparing the locomotor output in CP children with both TD toddlers and older TD children.

Despite inter-stride and inter-subject variability, walking speed monotonically increased with age, in agreement with the developmental growth of the body height. Gait parameters were on average comparable across different groups of children (hemiplegic, diplegic and older TD children), though children with diplegic CP showed on average a slower walking speed and shorter stride length in comparison with older TD children (*p* < 0.05, Tukey HSD). The vertical ground reaction forces, recorded in a subset of children (see Materials and Methods), often showed a decreased second peak in late stance in CP (Figure 1B), consistent with a previous study (Williams et al., 2011).

### Gait kinematics

**Figure 2:**
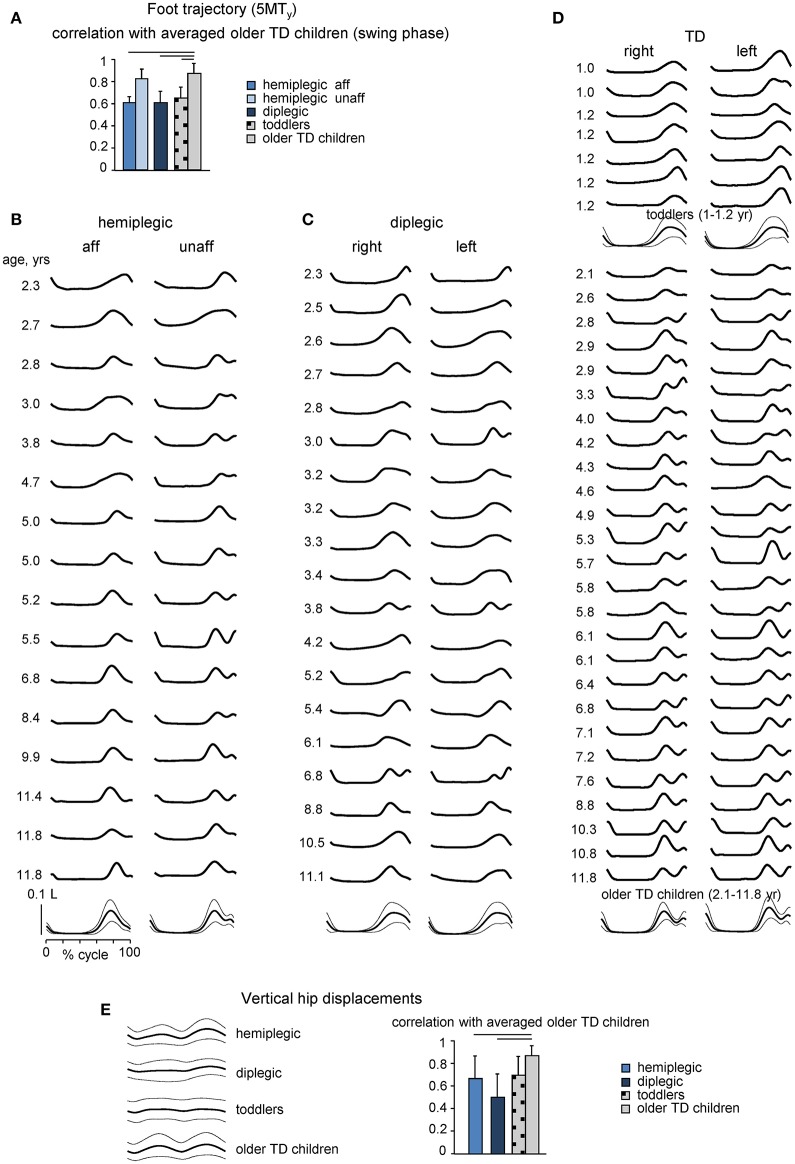
**Foot trajectory and vertical hip displacement characteristics**. **(A)**, Correlation coefficient (mean + SD) between vertical foot displacements (5MT_y_, swing phase) in children with CP and TD toddlers and ensemble average in older TD children. **(B–D)** Averaged across all strides individual foot trajectories (5MT_y_, ordered according to age from top to bottom) for children with hemiplegia (more affected and least affected sides), diplegia (right and left sides) and TD (right and left sides) children. TD children were divided into two subgroups: 7 toddlers aged 1–1.2 years and 26 older children aged 2.1–11.8 years. The lower curves in B-D illustrate ensemble-averaged (across subjects, ± SD) foot movements. 5MT_y_ is expressed in relative units (normalized by the limb length L). **(E)** Vertical hip (GT_y_, averaged for right and left legs) displacements averaged across subjects (mean ± SD, left panel) and correlation coefficients between GT_y_ data in children with CP and TD toddlers and corresponding ensemble averaged GT_y_ in older TD children (right panel). Horizontal lines denote significant differences with older TD children.

Foot trajectory characteristics and inter-segmental coordination differed systematically in children with CP as compared with those in age-matched TD children. Figures 2B–D shows the stride-averaged foot trajectory (the time course of the vertical foot displacements) for each participant (in ascending order of age) in hemiplegic, diplegic and TD children, respectively. The averaged template of foot motion is illustrated on the bottom. TD toddlers frequently moved the leg in such a way that the foot lift had only one maximum at mid-swing, while older TD children developed the adult-like pattern with two peaks and a minimum foot clearance during mid-swing (Figure 2D), consistent with previous studies (Ivanenko et al., 2005b; Dominici et al., 2007). Instead, children with CP (hemiplegic on the most affected side, diplegic on both sides) generally showed a one-peak foot path (Figures 2B,C), like TD toddlers. As a result, the correlation coefficient of the time course of the vertical foot displacement (5MT_y_) during swing in each group of participants with the ensemble average in older TD children was significantly smaller in diplegic and affected-side hemiplegic children with CP (0.67 ± 0.11 and 0.67 ± 0.03, respectively), as well as for TD toddlers (0.63 ± 0.04) (Figure 2A).

Many changes of gait parameters occur progressively in TD children enabling the child to assume a more adult-like style of walking (Hausdorff et al., 1999; Adolph et al., 2003). In older TD children, like in adults, the hip vaults over the stance leg as an inverted pendulum (Ivanenko et al., 2004). Accordingly, the temporal profile of vertical hip displacement (GT_y_) exhibits two peaks over each gait cycle, in coincidence with mid-stance of the right and left legs (Figure 2E). In TD toddlers, hemiplegic and diplegic children with CP, GT_y_ oscillations were variable from step to step and their mean profile differed relative to older TD children (correlation coefficients 0.66 ± 0.22 and 0.49 ± 0.23 for hemiplegic and diplegic children with CP, respectively, with the averaged profile of older TD children).

**Figure 3:**
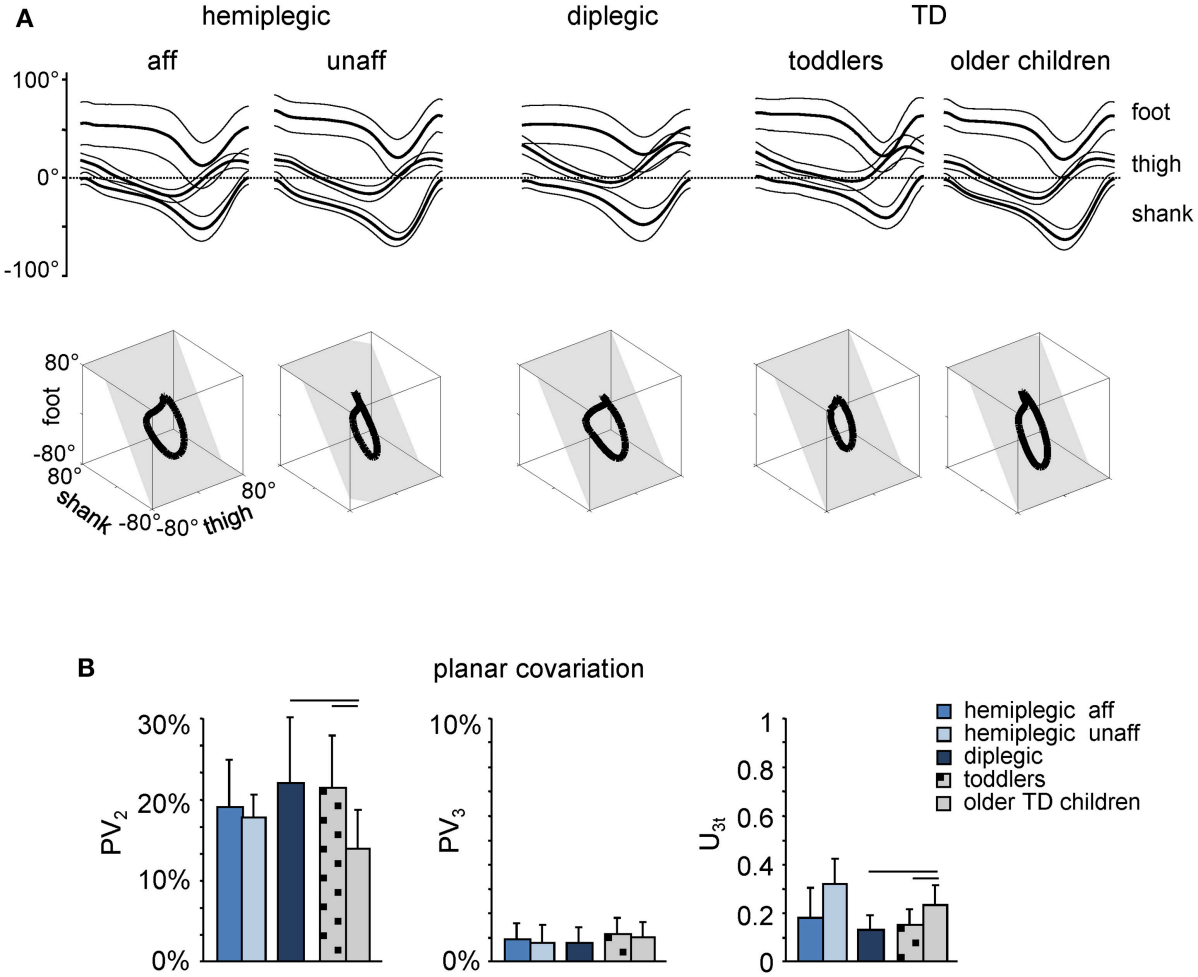
**Planar covariation of limb segment elevation angles during walking**. **(A)** Ensemble-averaged (mean ± SD) foot, thigh and shank elevation angles plotted vs. normalized gait cycle. As the relative duration of stance varied across strides and subjects, a hatched region indicates an amount of variability in the stance phase duration. Examples of 3-dimensional gait loops and interpolation planes are shown on the bottom from left to right for one child with hemiplegia (9.9 years) and one diplegia (8.8 years), as well as for one toddler (1.1 year) and one TD child (6.2 years). Gait loops are obtained by plotting the thigh waveform vs. the shank and foot waveforms (after mean values subtraction). Gait cycle paths progress in time in the counter-clockwise direction, touch-down and toe-off phases corresponding roughly to the top and bottom of the loops, respectively. The interpolation planes result from orthogonal planar regression. **(B)** Percentage of total variation explained by 2nd and 3rd principal components (PV_2_ and PV_3_, respectively) and u_3t_ parameter that characterizes the orientation of the normal to the plane are indicated for each group of children (mean ± SD). Horizontal lines denote significant differences with older TD children.

The inter-segmental coordination was assessed using the principal component analysis of limb segment elevation angles (see Materials and Methods). The top panels in Figure 3A illustrate ensemble-averaged (±SD) thigh, shank, and foot elevation angles in hemiplegic, diplegic, and TD children. In all children, temporal changes of the elevation angles of lower limb segments covaried along a plane, describing a characteristic loop over each stride (see examples in the lower panels of Figure 3A). Paths progress in time in the counter-clockwise direction along the loop, lift-off and touchdown corresponding approximately to the top and bottom of the loop, respectively. Planarity was quantified by the percentage of variance accounted for by the third eigenvector (PV_3_) of the data covariance matrix: the closer is PV_3_ to 0, the smaller the deviation from planarity. In all groups, PV_3_ was small (1–2%, Figure 3B, middle panel). The gait loop and its associated plane depend both on the amplitude and phase of the limb segment oscillations. The percentage of variance accounted for by the second eigenvector (PV_2_) was significantly greater in children with diplegia (21 ± 10%), and in TD toddlers (20 ± 10%) than in older TD children (13 ± 5%, *p* < 0.001, Tukey HSD), indicating a wider gait loop. The orientation of the covariance plane (u_3t_) was also significantly different in children with diplegia and TD toddlers with respect to older TD children (*p* < 0.0002, Figure 3B, right panel), consistent with previous observations (Cheron et al., 2001b; Dan et al., 2004).

### EMG patterns

**Figure 4:**
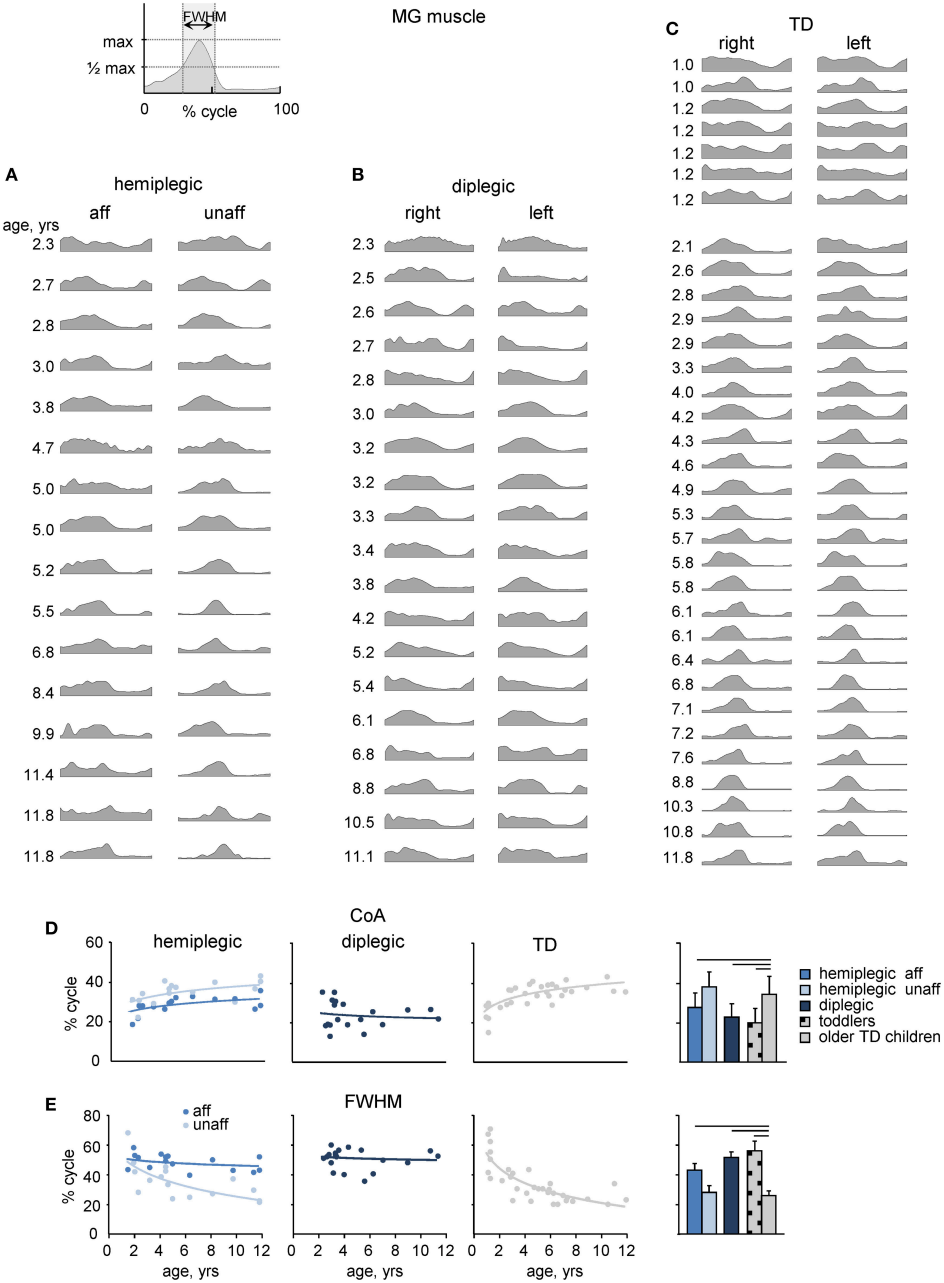
**MG muscle activity. (A–C)** Stride-averaged MG muscle activity for each subject (ordered by age) in children with hemiplegia, diplegia and TD children, respectively. **(D)** Center of activity (*CoA*) of the MG muscle in individual subjects as a function of age (left panels) and averaged across children (right panel). Continuous lines on the left panels represent exponential fittings. **(E)**, *FWHM* of MG activity as a function of age (left panels) and averaged across children (right panel). *FWHM* was calculated as the duration of the interval (in percent of gait cycle) in which EMG activity exceeded half of its maximum (see insert on the top). Horizontal lines denote significant differences compared with older TD children. Note a monotonic shift of the *CoA* toward later stance and a monotonic decrement of the *FWHM* in TD children with age, a lack of these changes in children with diplegia and their differential maturation on the affected and least affected sides in children with hemiplegia.

**Figure 5:**
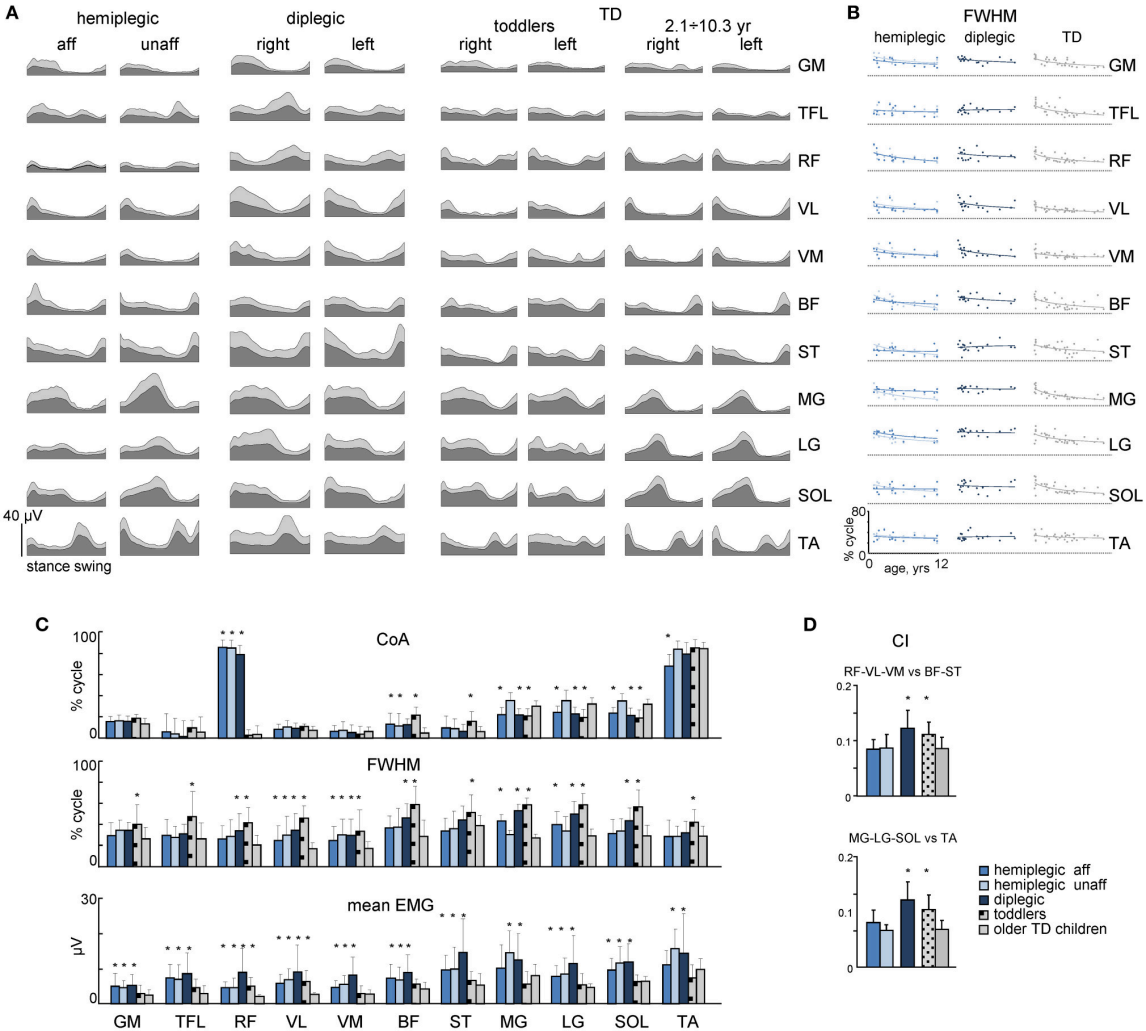
**Characteristics of EMG activity. (A)** Ensemble averaged (mean + SD) EMG activity patterns of 22 bilateral leg muscles recorded in children with CP and TD children. EMG data are plotted vs. normalized gait cycle. **(B)** *FWHM* of EMGs as a function of age in children with hemiplegia, diplegia and TD children. Continuous lines represent exponential fittings. Note a monotonic decrement of *FWHM* in TD children. **(C)** *CoA*, *FWHM* and mean leg muscle EMGs (means + SD) for children with CP and TD children. **(D)** Co-activation index (*CI*) for RF-VL-VM vs. ST-BF and MG-LG-SOL vs. TA pairs of antagonist muscles. Asterisks denote significant differences with older TD children.

We recorded EMG signals from 11 pairs of bilateral lower limb muscles. An example of age-related changes in the EMG pattern of a representative muscle (MG) in all participants is illustrated in Figures 4A–C, and the ensemble-averaged EMGs of all recorded muscles in children with CP and TD children are illustrated in Figure 5A. Despite inter-individual variability, there were systematic differences in the EMG activity between children with CP and TD children. Thus, in most children with CP and TD toddlers, there was prominent activity in the ankle extensors (MG, LG, SOL) throughout the stance phase starting with early stance, whereas activity in these muscles was normally observed during late stance in older TD children (Figures 4, 5). The activity of TA showed only one major peak at the beginning of swing in children with CP (hemiplegic, affected side, and diplegic) and TD toddlers with respect to two prominent peaks of older TD children (Figure 5A).

To characterize differences in the duration and timing of EMG activity between children with CP and TD children, we computed the *FWHM* and *CoA* (see Materials and Methods). In TD children, there was a clear developmental trend in the characteristics of EMG activity with age, which consisted in the reduction of the duration of EMG bursts and the adjustment of their timing (Figures 4C, 5B). Indeed, the major bursts of activity of most muscles (hamstrings, quadriceps, ankle plantar-flexors) tended to be wider in children with CP and TD toddlers with respect to older TD children. Also, the total mean level of activation of leg muscles in children with CP (expressed in μV, Figure 5A) tended to be higher with respect to that in older TD children (Figure 5C bottom), although differences in EMG intensity may reflect potential differences in skin impedance between subjects. Moreover, we examined the *FWHM* as a function of age (Figure 5B). The analysis revealed that *FWHM* decreased systematically with age in most muscles in TD children. In contrast, this age-related trend was limited or absent in several muscles in children with diplegia and hemiplegia (on the affected side, Figure 5B). The *CoA* systematically shifted to the earlier phases of stance in distal muscles (MG, LG, SOL) for diplegic children, affected-side hemiplegic children and TD toddlers with respect to older TD children (*p* < 0.001; Figure 5C, upper panel). Finally, children with diplegia and TD toddlers showed significantly higher co-activation index values throughout the gait cycle both for RF-VL-VM vs. ST-BF and MG-LG-SOL vs. TA pairs of antagonist muscles with respect to older TD children (Figure 5D).

### Basic muscle activation patterns

**Figure 6:**
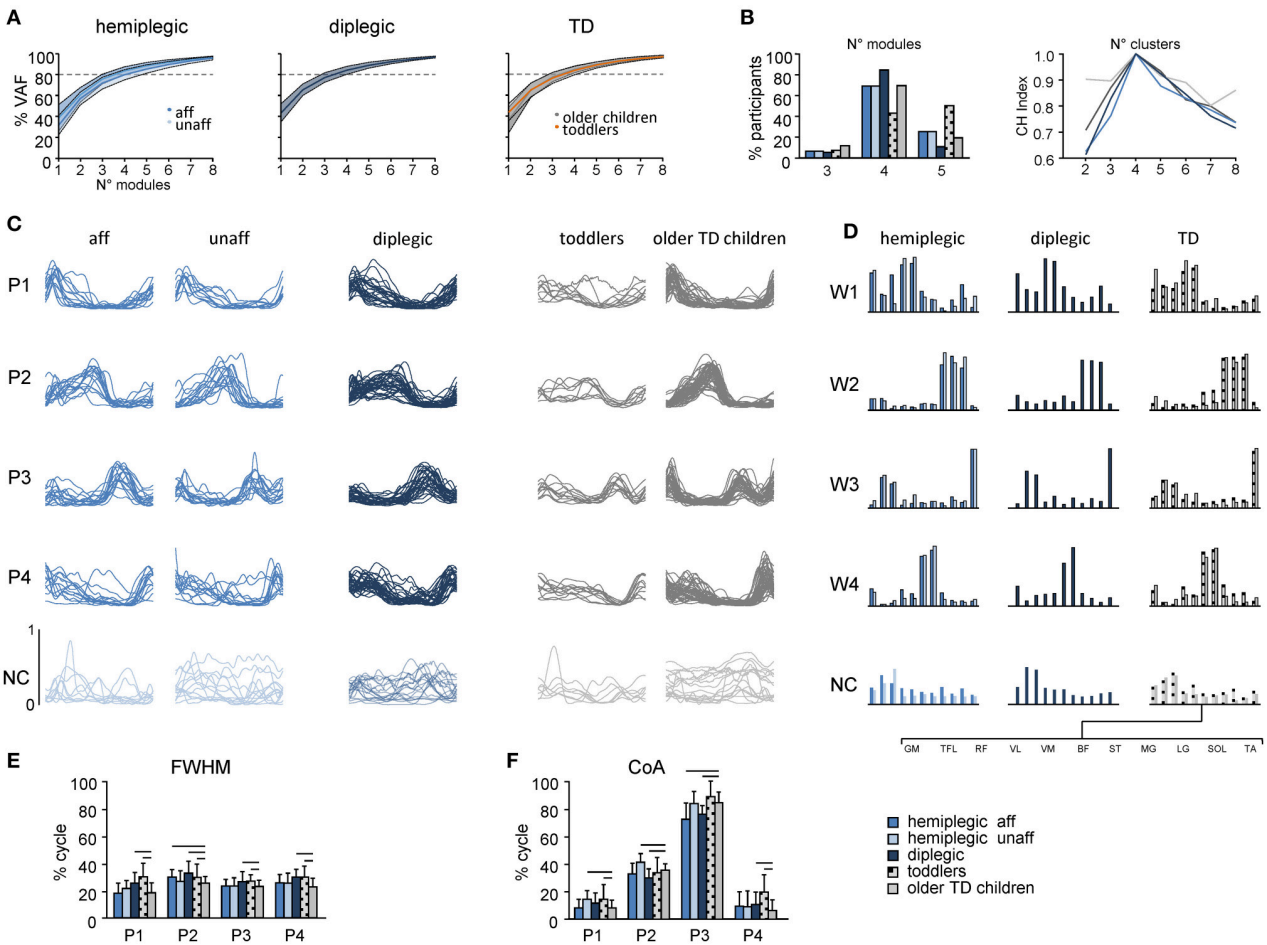
**Statistical analysis of EMG patterns using non-negative matrix factorization**. The data for the most affected and less affected sides of children with hemiplegia and for TD toddlers and older children are shown separately. **(A)** Cumulative percent of variance (*VAF*) (±SD) explained by basic EMG components in hemiplegic (left), diplegic (center) and TD (right) children. **(B)** The number of modules needed to account for cycle-by-cycle variability of muscle activity estimated using the “best linear fit” method (left) and the normalized CH index (Caliñski and Harabasz, 1974) to evaluate the number of clusters in each group (right). Even if three to five modules were sufficiently representative in a few children, EMG activity in most children was well accounted for by four modules (>80% of VAF, **A**). **(C)** Comparison of muscle module (basic activation patterns) structures across different groups. Each curve represents the mean (across strides) pattern for an individual child. Common (across children) basic patterns were plotted in a “chronological” order (with respect to the timing of the main peak) and modules were ranked based on their best similarities (see Materials and Methods). Note that synergies with low structural consistency across children (NC, Not Classified) were plotted separately on the bottom in toned-down colors. **(D)** Group mean weights (synergies). **(E,F)** mean (+SD) *FWHM* and *CoA* of consistent basic activation patterns (P1–P4). Horizontal lines denote significant differences compared with older TD children. Note wider P2 and P4 patterns in children with CP and toddlers with respect to older children **(E)**, consistent with wider EMGs (Figure 5C, bottom).

The analysis of dimensionality using the non-negative matrix factorization method showed that EMG activity changes during walking are adequately captured by a small number of motor modules in all groups (Figures 6A,B). Even though three to five modules were sufficiently representative in a few participants, EMG activity in most participants was well accounted for by four modules using the “*Best linear fit*” method (Figure 6B, left panel). The cluster evaluation criterion showed that the number of motor modules that maximizes the CH index (Caliñski and Harabasz, 1974) for each group (i.e., the number of modules similar across participants) was four (Figure 6B, right panel). Based on this number, the basic activation patterns and the corresponding weightings (muscle synergies) were grouped and reported in Figures 6C,D. The muscle weightings and pattern activation timing were qualitatively similar between groups. Each basic pattern peaked at a particular time of the gait cycle and showed specific characteristics. Pattern P1 mainly loaded on GM, TFL, RF, VL, and VM muscles and peaked around touchdown, providing body support during weight acceptance. Pattern P2 mainly loaded on the ankle plantar-flexor muscles (MG, LG, and SOL) and contributed to forward body propulsion. Pattern P3 was primarily related to the activity of TA for foot lift, and to a lesser extent TFL and RF during swing. Pattern P4 was mainly involved in the modulation of hamstrings (BF and ST) for leg deceleration during late swing and early stance. Unmatched modules were isolated and pooled together as Not Classified (Figures 6C,D).

Despite a comparable structure of motor modules in children with CP and TD children (Figures 6A–D), we found significant differences in their quantitative characteristics (Figures 6E,F). We report statistics considering only the first four basic patterns, which were the most consistent (Figure 6C). *FWHM* was significantly greater for P2 in children with CP and TD toddlers with respect to older TD children (*p* < 0.05, Figure 6E), reflecting a similar widening of the EMG envelopes (Figure 5C), and significantly greater for P1, P3, and P4 in diplegic and TD toddlers. The *CoA* of basic patterns was generally similar for the children with CP and TD children, however we found that P1 and P2 shifted to slightly later phases of the gait cycle for the less affected side of children with hemiplegia, P2 and P3 shifted to earlier phases in children with diplegia and in the affected-side of children with hemiplegia, P4 shifted to a later phase in children with diplegia and TD toddlers compared with TD children (Figure 6F).

While the analysis reported in Figure 6 was performed on normalized EMG data, we remark that the emerging structure of coordinated muscle activity did not depend significantly on whether or not the data were normalized. Indeed, when we analyzed EMGs in microvolts, we found that the number of clusters was still four for all groups of participants and pattern characteristics showed an increase of *FWHM* in children with CP and toddlers.

### Spinal segmental motoneuron output

**Figure 7:**
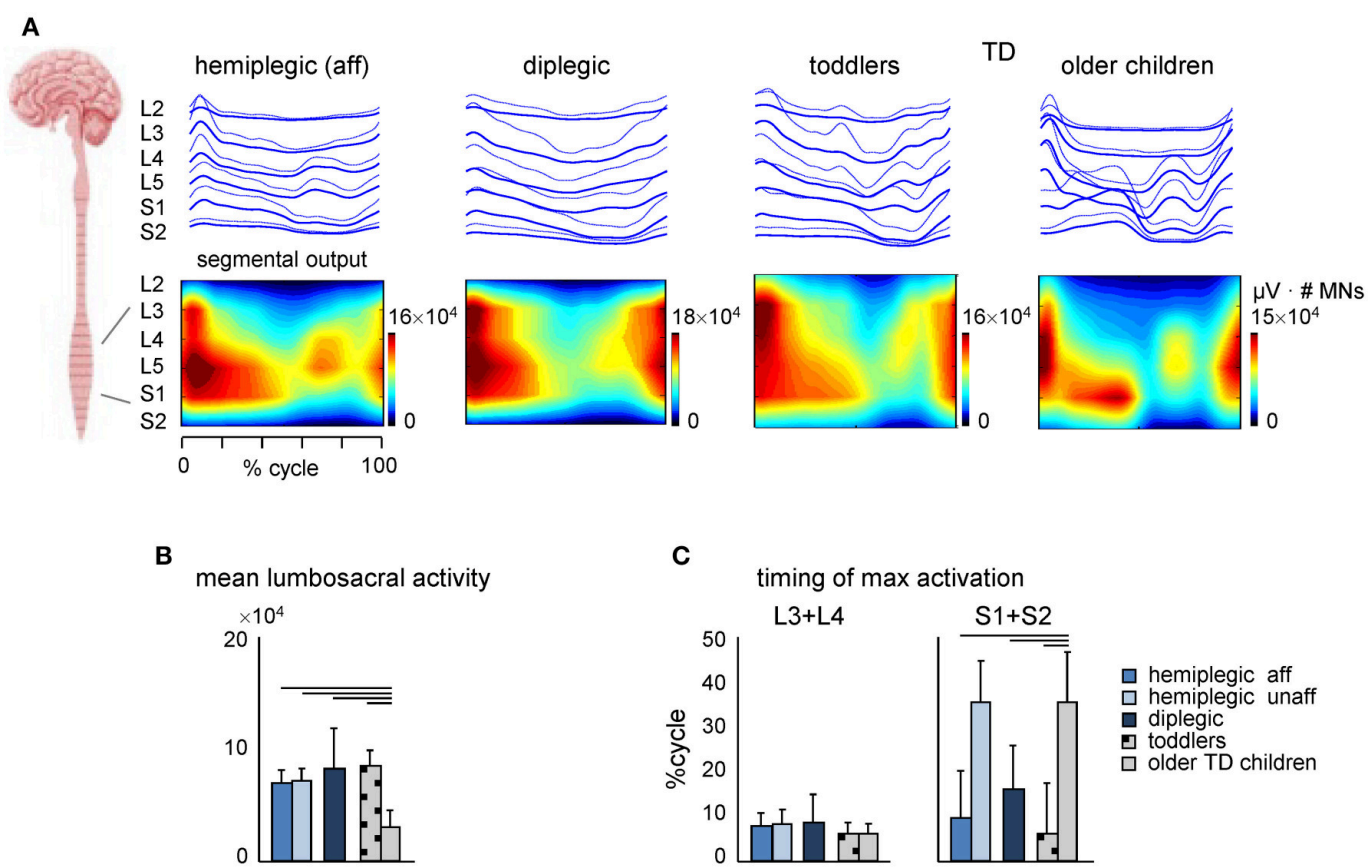
**Spatiotemporal maps of motoneuron activity of the lumbosacral enlargement in children with CP and TD children. (A)** Output pattern of each segment is shown in the top panels (thick traces, means; thin traces, means + 1SD), while the same pattern is plotted in a color scale (using a filled contour plot) at the bottom. Motor output (averaged across children, reported in units of number of MNs) is plotted as a function of gait cycle and spinal segment level (L2 − S2). **(B)**, Depicted are mean (+SD) activation of lumbosacral activity (averaged across both gait cycle and spinal level). **(C)** Timing of maximum activation of lumbar (L3 + L4) and sacral (S1 + S2) segments. The values represent the mean + SD. Lines over bars denote significant differences compared with older TD children.

Figure 7A shows the average segmental motoneuron output over the step cycle obtained by mapping the EMG activity profiles onto the rostrocaudal location of the motoneuron pools in the lumbosacral enlargement. Figures 7B,C illustrate quantitative characteristics (intensity and timing) of the spatiotemporal activation of MNs in children with CP and TD children. Even though we recorded a limited set of muscles, we have previously shown that the muscles recorded here are those that contribute mostly to the overall spinal maps (Ivanenko et al., 2013a; La Scaleia et al., 2014). Furthermore, the recorded muscles (Figure 5A) contribute a large part of the total cross-sectional area of leg muscles (Ward et al., 2009).

The prominent feature of these maps in older TD children (Figure 7A) was a distinct activation of lumbar and sacral segments during early and late stance, respectively. This activation pattern in older TD children appears to be a precursor closely related to the mature pattern seen in young healthy adults, where the activations are shorter and with an evident separation of the distinct bursts (Monaco et al., 2010; Ivanenko et al., 2013a).

**Figure 8:**
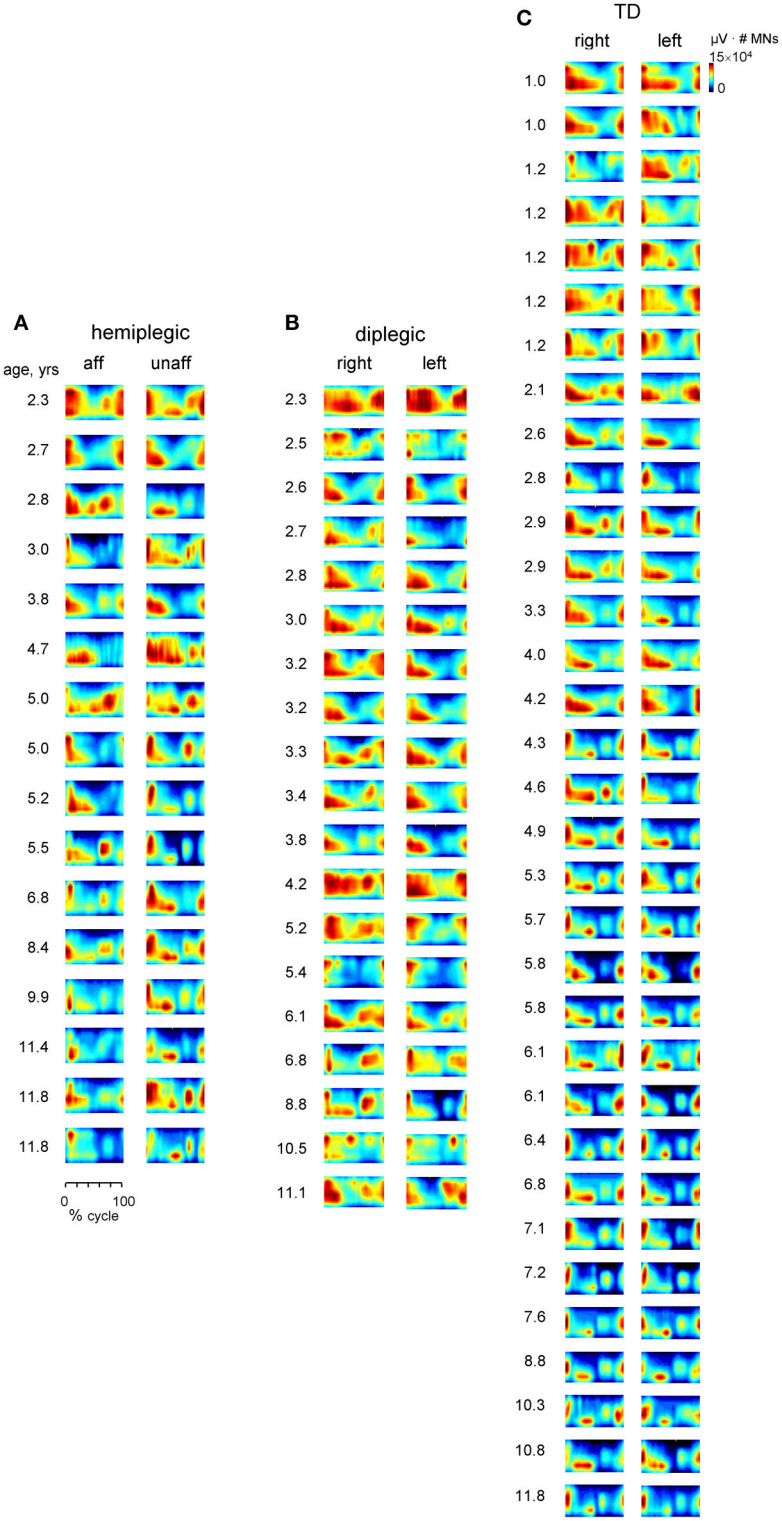
**Spatiotemporal maps of motoneuron activity of the lumbosacral enlargement for each subject (ordered by age) in children with hemiplegia (A), diplegia (B), and TD children (C)**. The pattern is plotted in a color scale (using a filled contour plot) for both sides of the spinal cord. Motor output (averaged across strides, reported in units of μV × number of MNs) is plotted as a function of gait cycle and spinal segment level (L2-S2).

In contrast, despite inter-individual variability (see individual spinal maps, Figure 8), the dominant feature of the maps in TD toddlers and in children with CP (diplegic and affected-side hemiplegic) was a similar timing of the maximum activity of lumbar and sacral segments at the onset of the stance phase, consistent with their quasi-synchronous involvement (Figure 7C). As a result, the timing of maximum activity of sacral segments was significantly different from that in older TD children (*p* < 0.001, Tukey HSD), while the timing of lumbar segments did not show any significant difference between the groups of children (Figure 7C). It is also worth noting that differences in the biomechanics of stepping (e.g., toe-walking in some children) cannot fully account for the specific motor patterns in children with CP (Figure 7), since tiptoe walking in adults still results in a more segregated structure with separate lumbar and sacral activations (La Scaleia et al., 2014). In addition, the total intensity of the MN output of the lumbosacral enlargement was significantly smaller in older TD children (Figure 7B), consistent with lower intensity and narrower EMG bursts in these children (Figure 5C). Furthermore, the main features of CP spinal maps did not change significantly if we assumed slightly different coefficients in the Equation (7) or when using the Sharrard data table. In both cases, the timing of maximum activity of sacral segments was also significantly different in CP and TD toddlers from that in older TD children (*p* < 0.001, Tukey HSD), as well as the total intensity of the MN output was smaller in older TD children.

## Discussion

The results are consistent with the hypothesis that maturation of the spinal locomotor output is impaired in children with CP. Although we did not study longitudinal changes in the locomotor patterns, the wide age range of our sample of participants clearly showed that the spinal motor output in children with CP remains similar to that of much younger TD children and lacks the age-related changes present in TD children (Figures 2–7). In particular, we found that children with CP lack maturation of the foot trajectory control and intersegmental coordination (Figures 2, 3), muscle activity patterns (Figures 4–6) and the spatiotemporal characteristics of the spinal segmental output (Figure 7). It is also worth noting that children with hemiplegic CP showed differential maturation of these features on the affected and less affected lower limbs.

### Maturation of gait kinematics

Natural maturation of the neuromuscular apparatus, motor learning and environmental enrichment (Sale et al., 2014) play important roles during development. Progressive changes of gait kinematics and kinetics may depend on musculoskeletal growth (including foot shape modifications and ossification of the soft bones of the feet, Bertsch et al., 2004), development of the vestibular system (Wiener-Vacher et al., 1996), decrease of central conduction delays (Eyre et al., 1991) and muscle contraction time (Dayanidhi et al., 2013), and maturation of central neuronal pathways (Paus et al., 1999). In TD toddlers at their first unsupported steps, stable planar covariation of the limb segment angular motion (Cheron et al., 2001b), foot trajectory (Dominici et al., 2007) and pendulum-like behavior of the COM (Ivanenko et al., 2004) are still immature. They co-evolve toward mature values within a few months, though anthropometrical changes and developmental tunings continue for many years. Children with CP show difficulties in developing the major features of adult gait, such as ankle dorsiflexion at heel strike, knee flexion in mid-stance, and ankle plantarflexion with hip extension at the end of stance (Berger et al., 1982, 1984; Leonard et al., 1991; Berger and Adolph, 2007). They often place the foot flat or on the toes, show anticipated activation of the calf muscles, an increase in antagonist coactivation of the leg muscles, low activation of the gastrocnemius muscle, impaired ability of tibialis anterior to dorsiflex the ankle during swing, and enhanced short latency proprioceptive reflexes (Berger et al., 1982, 1984).

Many gait features idiosyncratic of TD toddlers characterize the gait of children with CP throughout childhood. In particular, a characteristic feature of CP gait, reminiscent of TD toddlers, is the prominent single-peak foot lift during swing (Figures 2A–D) and disordered vertical hip displacements (Figure 2E). The latter finding is consistent with a reduced capacity in absorbing and decelerating the speed of the center of mass and pendular energy transduction in CP (Zollinger et al., 2016). The authors argued that this may have an important implication in rehabilitation programs that assess and train gait in adolescents with CP. Indeed, the gravity-related pendulum mechanism of walking is present in TD children (Cavagna et al., 1983), nevertheless, it does not seem to be implemented at the onset of independent walking (Ivanenko et al., 2004) as well as young children have a lower efficiency of positive muscular work production than adults during walking (DeJaeger et al., 2001; Schepens et al., 2004). Given that this mechanism is effective in decreasing the walking energy cost, gait training program with real-time feedback of the body's center of mass vertical displacement (Massaad et al., 2010) may be complementary to current concepts in rehabilitation of gait in children with CP.

The stereotyped, two-peaked trajectory of the foot with minimal toe clearance at mid-swing represents the result of a safe, accurate endpoint control that depends on multi-segmental coordination in both the stance and swing limbs (Bernstein, 1967; Winter, 1992; Ivanenko et al., 2002). This behavior was not observed in children with CP; instead, a single-peaked foot lift was observed across all sampled ages (Figure 2), as was the immature characteristics of the inter-segmental coordination (the shape of the gait loop and its orientation, Figure 3B). The wider gait loop in children with diplegia and TD toddlers (larger PV_2_, Figure 3B) may be related to the phasing between thigh and shank angular motion, as a mechanism to facilitate the increase in toe clearance (MacLellan et al., 2011).

However, despite the similarities, the gait in children with CP cannot be equated to that in TD toddlers, since in addition to the observed lack of maturation of the spinal locomotor output, children with CP may develop other abnormalities due to impaired corticospinal interactions, including dystonia, muscle contractures, incoordination (Crenna, 1998; Gormley, 2001). For instance, early works suggest that cortical control is needed for accurate foot placement (Forssberg, 1985; Beloozerova and Sirota, 1993), so that particular difficulty in children with CP may be attributable to abnormal development in the motor cortex and/or the corticospinal tract (CST). Also, we know that the muscles are weak and often quite atrophic, so their response and the final output as measured by EMG and kinematics may not be just due to neurophysiological but also to biomechanical and histopathological changes (Hanson and Jones, 1989; Sutherland and Davids, 1993).

### Maturation of spinal locomotor output

In healthy people, lower limb muscle activation for walking involves multiple neural networks that are tightly interconnected. It has been suggested that the locomotor patterns in children with CP depend on a disruption of appropriate supraspinal signals controlling afferent and efferent information at the spinal level (Berger, 1998). Although the rhythmic part of the modulation pattern is present in less severe CP, there often is no significant tonic reflex depression with age, reflecting a lack of maturation of the CST (Myklebust, 1990; Hodapp et al., 2009; Achache et al., 2010). Thus, the functional organization of spinal circuitry is likely impaired before children with CP start walking. The previous studies also suggest that interlimb locomotor coordination in CP may depend mostly on the coupling between spinal pattern generators, coordinated by brainstem mechanisms, rather than primarily on cortical structures (Meyns et al., 2014). Nevertheless, the impaired “state” of the spinal circuitry in these children can essentially affected their gait. Therefore, investigating the spinal locomotor output may provide further insights into the functioning of spinal pattern generators in CP.

We evaluated the organization of the spinal locomotor output by analyzing regularities in the coordinated muscle activation patterns (Figure 6) and by mapping the activity patterns from simultaneously recorded muscles onto the anatomical localization of the corresponding MN pools derived from published myotomal charts (Figure 7). Our results demonstrated a similar modular structure of the motor output in CP and TD children, but children with CP had wider basic temporal activation patterns (Figure 6). EMG activity in most TD and children with CP was well accounted for by four modules (Figures 6A,B). The smaller number of muscle synergies found in children with CP in some previous studies (Steele et al., 2015; Tang et al., 2015; Shuman et al., 2016) may be a consequence of the criterion used to define the minimum number of synergies sufficient to account for overall EMG activity (Hug et al., 2012; Russo et al., 2014) and/or the limited number of recorded muscles (Steele et al., 2013; Zelik et al., 2014; Damiano, 2015). Nevertheless, it is worth noting that the observed phenomenon of widening of basic activation patterns (Figure 6) does not depend on the exact number of modules retained by the specific non-negative matrix factorization procedure (Martino et al., 2015). The increased co-activation index of antagonist muscles observed in children with diplegia and TD toddlers (Figure 5D, see also Damiano et al., 2000; Prosser et al., 2010) could be related at least in part to EMG widening.

The co-activation pattern in children with CP can partially be attributed to the leg configurations with slightly bended knee joints. Nevertheless, widening was observed in the EMG envelopes of numerous muscles (Figure 5) and corresponding basic activation patterns (Figure 6E), as well as the cause-and-effect relationship is open to discussion. For instance, producing heel strike with a straightened leg represents a unique feature of human gait, is not a simple task and requires learning and accurate timing of muscle activation. Therefore, slightly bended knee joints and the lack of an adult-like heel-to-toe rolling pattern during stance in children with CP can be a result of wider EMG bursts. Broader activation patterns likely imply higher metabolic cost and may also limit adaptation to different walking conditions and coordination with voluntary movements that require appropriate activation timings/duration (Ivanenko et al., 2005a). The potential mechanism for wider EMG bursts may be related in part to spasticity or impairments in muscle tone in CP.

The current data do not allow differentiating the contribution of descending supraspinal vs. spinal influences to the observed phenomenon of EMG burst widening. Likely, both networks are important considering an impaired state of the spinal circuitry in CP and an essential and more important role of the supraspinal motor areas in human walking than in animals (Yang and Gorassini, 2006). The functional link between EMG patterns and the compromised inter-segmental coordination (Figures 2, 3) may also be grounded on the oscillation concept (Cheron, 2015) relating the rhythmic patterning elements. The inter-limb phase and the inter-segmental coordination pattern can be controlled by symmetrically organized unit burst generators for each joint, limb segment, or groups of muscles (Grillner, 1981) and may emerge from the coupling of neural oscillators with limb mechanical oscillators (Lacquaniti et al., 2012b). In fact, the inter-segmental coordination rule can be modeled by simple oscillators coupled with appropriate time shifts (Barliya et al., 2009) and implemented in a dynamic recurrent neural network mimicking the natural oscillatory behavior of human locomotion (Hoellinger et al., 2013). Thus, the impaired characteristics of the inter-segmental coordination (the shape of the gait loop and its orientation, Figure 3B) and changes in activation timings (Figures 5C, 7C) may be indicative of a possible alteration of the oscillatory commands in children with CP. Finally, the immature developmental state of the cerebellum (Vasudevan et al., 2011) may also contribute, since wider EMG bursts have been previously reported in cerebellar patients (Hallett et al., 1975; Martino et al., 2014). Whatever the exact mechanism for wider bursts of muscle activity, it represents the characteristic feature of spinal motoneuronal output in CP (Figures 4–7).

The optimization of timing and amplitude of muscle activity represents an important feature of motor patterns development (Forssberg, 1985; Hadders-Algra et al., 1992; Lacquaniti et al., 2012a). The functional reorganization of the spinal motor output during early development consists in a gradual shift of the timing of stance related muscle activity and corresponding activity of the sacral segments of the spinal cord (Ivanenko et al., 2013a; Figure 7), and a gradual decrease of the EMG burst duration with age (Figures 4, 5B,C): the older the child, the closer the waveform to the adult. These age-related adjustments of neuromuscular control were largely absent in our sample of children with CP until 11–12 years (Figures 4–7) and muscle activity remained similar to those of TD toddlers (Figures 5C, 6), consistent with the hypothesis of immaturity of CP locomotor networks.

## Concluding remarks

In addition to the physiological relevance of investigating early development of the spinal locomotor circuitry (Dominici et al., 2011), the present results may have important clinical implications. The development of efficient and independent walking is an important therapeutic goal for many children with CP (Willoughby et al., 2009; Smania et al., 2011; Degelean et al., 2012; Graham et al., 2016). Given a positive effect of repetitive locomotor exercise on gait characteristics in children with CP (Smania et al., 2011), the rehabilitative protocol may further focus on improving the structure of the spinal locomotor output, e.g., by providing a feedback on the basic activation pattern burst durations. New brain stimulation approaches (e.g., Gerasimenko et al., 2015) may be applied as well in children with CP in order to attempt restoring improved functions of the locomotor network. There may also be critical developmental windows (for language, transition to bipedalism, etc.) during which specific experiences have a greater effect on the developmental process than at other times (Ivanenko et al., 2013b; Yang et al., 2013). For instance, it is likely that, similar to cats (Salimi et al., 2008; Friel et al., 2012), there is a critical period for the development of the human CST, which is presumably the last descending pathway to mature in children (Yakovlev and Lecours, 1967; Martin, 2005; Yeo et al., 2014). In human neonates and children aged less than 3 years, CST projection activities shape the spinal cord motor function (Eyre et al., 2001). Based on the timing of myelination of the CST, it could be estimated that the critical period for the establishment of appropriate effects of CST onto lumbosacral spinal circuitry corresponds to an age between few months and 2 years (Yang et al., 2013). The efficacy to repair CST connections to the spinal cord is strongly reduced after the critical period and is insufficient to restore significant function unless promoted (Friel et al., 2014). Thus, the lack of maturation of the spatiotemporal spinal locomotor output between 2 and 12 years in children with CP (Figures 2–7) may be consistent with the idea of critical developmental windows (Hadders-Algra, 2004; Friel et al., 2014), so that intensive training in children with CP younger than 2 years of age could enhance more effectively their walking function (Yang et al., 2013). The common feature of the spinal interneuronal networks is that input from several sources, including descending motor pathways and sensory afferents, is distributed to their constituent neurons, inferring interactions between neurons both within and between various populations (Hultborn, 2001; Jankowska, 2008). We do not know the specific neural substrates underlying the altered (or immature) output from the spinal motoneurons in children with CP, and only future studies will help clarify this issue.

## Author contributions

GC, YI, MM, DM, and FL have conceptualized the study and designed the experimental protocols; AS and DM, evaluation and selection of children with cerebral palsy; GC, MM, AS performed the experiments; GC, MM, and GM performed the data analysis. All the authors made contributions in drafting the manuscript and interpreting the results and have approved the final version.

## Funding

This work was supported by the Italian Ministry of Health (IRCCS Ricerca corrente), Italian Space Agency (COREA grant 2013-084-R.0), and Horizon 2020 Robotics Program (ICT-23-2014 under Grant Agreement 644727-CogIMon).

### Conflict of interest statement

The authors declare that the research was conducted in the absence of any commercial or financial relationships that could be construed as a potential conflict of interest.

## Abbreviations

BF: biceps femoris (long head)
CH index: Calinski-Harabasz Index
CoA: center of activity
CP: cerebral palsy
EMG: electromyographic
FWHM: full width at half maximum
GM: gluteus maximus
GMFCS: Gross Motor Function Classification System
GMFM: Gross Motor Function Measure
GT: greater trochanter
L: limb length
LG: gastrocnemius lateralis
MG: gastrocnemius medialis
MN: motoneuron
Pi: basic activation pattern
PVi: percentage of variance accounted for by the i-th eigenvector
RF: rectus femoris
SOL: soleus
VL: vastus lateralis
ST: semitendinosus
TA: tibialis anterior
TD: typically developing
TFL: tensor fascia latae
VAF: variance accounted for
VM: vastus medialis
W: weighting coefficient (muscle synergy)
5MT: fifth metatarso-phalangeal joint.
